# Evaluation of Three Sampling Methods to Monitor Outcomes of Antiretroviral Treatment Programmes in Low- and Middle-Income Countries

**DOI:** 10.1371/journal.pone.0013899

**Published:** 2010-11-10

**Authors:** Jean-Michel Tassie, Karen Malateste, Mar Pujades-Rodríguez, Elisabeth Poulet, Diane Bennett, Anthony Harries, Mary Mahy, Mauro Schechter, Yves Souteyrand, François Dabis, for the ART Linc of IeDEA and MSF collaborations

**Affiliations:** 1 Department of HIV/AIDS, World Health Organisation, Geneva, Switzerland; 2 INSERM U857 and Institut de Santé Publique, Epidémiologie et Développement (ISPED), Université Victor Segalen, Bordeaux, France; 3 Epicentre, Paris, France; 4 Centers for Disease Control and Prevention, Atlanta, Georgia, United States of America; 5 International Union Against Tuberculosis and Lung Disease, Paris, France; 6 London School of Hygiene and Tropical Medicine, London, United Kingdom; 7 The Joint United Nations Programme on HIV/AIDS (UNAIDS), Geneva, Switzerland; 8 Universidade Federal do Rio de Janeiro, Rio de Janeiro, Brazil; University of Cape Town, South Africa

## Abstract

**Background:**

Retention of patients on antiretroviral therapy (ART) over time is a proxy for quality of care and an outcome indicator to monitor ART programs. Using existing databases (Antiretroviral in Lower Income Countries of the International Databases to Evaluate AIDS and Médecins Sans Frontières), we evaluated three sampling approaches to simplify the generation of outcome indicators.

**Methods and Findings:**

We used individual patient data from 27 ART sites and included 27,201 ART-naive adults (≥15 years) who initiated ART in 2005. For each site, we generated two outcome indicators at 12 months, retention on ART and proportion of patients lost to follow-up (LFU), first using all patient data and then within a smaller group of patients selected using three sampling methods (random, systematic and consecutive sampling). For each method and each site, 500 samples were generated, and the average result was compared with the unsampled value. The 95% sampling distribution (SD) was expressed as the 2.5^th^ and 97.5^th^ percentile values from the 500 samples. Overall, retention on ART was 76.5% (range 58.9–88.6) and the proportion of patients LFU, 13.5% (range 0.8–31.9). Estimates of retention from sampling (n = 5696) were 76.5% (SD 75.4–77.7) for random, 76.5% (75.3–77.5) for systematic and 76.0% (74.1–78.2) for the consecutive method. Estimates for the proportion of patients LFU were 13.5% (12.6–14.5), 13.5% (12.6–14.3) and 14.0% (12.5–15.5), respectively. With consecutive sampling, 50% of sites had SD within ±5% of the unsampled site value.

**Conclusions:**

Our results suggest that random, systematic or consecutive sampling methods are feasible for monitoring ART indicators at national level. However, sampling may not produce precise estimates in some sites.

## Introduction

At the end of 2009, more than 5 million people were receiving antiretroviral therapy (ART) in low- and middle-income countries,[1] out of 33.4 million people (estimated range: 31.1 million–35.8 million) living with HIV [2], [3]. This represents a 30% increase in one year and a 13-fold increase in ART uptake in six years. Monitoring of ART programmes is critical for understanding when sites are under-performing and estimating the potential impact of treatment, at the population level and for program management at different levels of the health system. In addition, reporting of such indicators helps to sustain national and global commitment to monitor quality of care while expanding access to ART and its growing use.

Many countries are still struggling to report national programme indicators. In 2009, 70 out of 149 low- and middle-income countries (47%) reported statistics on patient retention on ART at 12 months and 30 (20%) at 48 months [4]. This outcome indicator is one of several core indicators recommended to monitor the Declaration of Commitment on HIV/AIDS during the United Nations General Assembly Special Session on HIV/AIDS (UNGASS) [5]. Although some countries have highly automated information systems, many ART sites within countries have difficulty in maintaining the registers/databases necessary to produce these statistics. A number of factors may explain the difficulties in generating good quality information. Many ART programmes are relatively recent, yet facing large and rapid increases in the number of patients starting therapy. They have thus logically focused their attention on putting new patients into care and reporting baseline information rather than on follow-up. In addition to ART, HIV care includes various other components (e.g. prevention and treatment of opportunistic infections including tuberculosis or integration with reproductive health services) that require regular monitoring. With the rapid expansion of ART services and decentralisation to peripheral health centres, data management has to occur at all levels of health service provision and should be designed to be as simple and user-friendly as possible in order for it to be adopted universally. Finally, ART treatment is life-long, leading to continued workload increase due to inclusion of new patients and continued follow-up.

In this paper we evaluated the performance of three sampling approaches to produce two clinic and higher level indicators at 12 months: retention on ART [6] and proportion of patients lost to follow-up, using existing databases from ART programmes in low- and middle-income countries.

## Methods

### Sources of data

ART-LINC of IeDEA is a large collaborative network of HIV/AIDS treatment programmes in low- and middle-income countries in Africa, South America and Asia, originally funded by the United States National Institutes of Health (Office of AIDS Research) and the French Agence Nationale de Recherches sur le Sida et les hépatites virales (ANRS). It is now part of the International epidemiological Database to Evaluate AIDS collaboration of the NIH (http://www.iedea-hiv.org). This network was established in 2003 to characterize the prognosis of HIV-infected patients treated with ART in resource-limited settings, to compare the experience between different settings, delivery modes and types of monitoring; and to compare outcomes with those observed in industrialized nations [7]. Staff at the sites filled in a detailed abstraction form using their own medical records, or downloaded the required data from their own electronic medical records (EMR) systems. Data were merged anonymously at the University of Bern, Switzerland and the University of Bordeaux, France [8].

Since 2001, Médecins Sans Frontières (MSF) has provided ART in 26 countries with a high HIV prevalence, most of them in Africa and Asia. Basic patient individual clinical, laboratory and treatment information are routinely collected at every clinic visit using standardized forms. Data are continuously entered into the Follow-up and Care of HIV Infection and AIDS (FUCHIA) EMR software (Epicentre, Paris, France). Capacity building and maintenance of the databases are funded by MSF and data centralized anonymously in Epicentre – Paris, France, the epidemiological support office of MSF [9], [10].

### Ethics statement

International review boards in each country have approved the use of routinely collected programme data at all ART-LINC sites. This study was approved by the research ethics review committee of the World Health Organisation and the ethics review board of Médecins Sans Frontières. The data analysed are primarily collected for patient management and program monitoring, with the agreement of the ministries of health of the countries. Because of this reason, patient informed consent is not routinely requested in all sites. All ethic boards were aware of this and approved the secondary use of data for this analysis.

### Selection of sites and indicators

For this analysis we selected all ART sites with more than 260 ART naive adults aged ≥15 years old starting ART in the year 2005. All databases were updated more than 12 months after the inclusion of the last patient in 2005. We analysed the patient status at 12 months, classified as followed on ART, dead, stopped treatment, and lost to follow-up (LFU). Patients LFU were defined as patients with no recorded visit for ≥90 days from the last visit within the first year. Patients transferred to another ART programme within the first 12 months of treatment were excluded from the analysis (n = 896 or 3% of the overall database).

We studied two indicators measured 12 months after ART initiation, the proportion of patients LFU and the proportion retained on ART.

### Sampling methods

For each ART site, the required sample size was calculated, assuming a retention at 12 months of 75% as n≥[(u^2^_α_*N*p*(1-p))/(Δ^2^ * (N-1)+u^2^_α_*p*(1-p))] with n sample size, N total population of each site, p expected proportion estimated at 75%, Δ precision estimated at 0.05 and u_α_ estimated at 1.96. We used the sample required to ensure that the 95% (i.e., 1-alpha) confidence interval of the 75% proportion had a width at least 2 times Delta where Delta is the required precision. Three sampling methods were considered: the random selection, systematic and consecutive sampling. In random sampling each patient of each clinic database had an equal probability of selection and each patient was selected using a random number allocation. In systematic sampling, the first patient was randomly selected and the others were drawn from the clinic database according to a sample interval defined as N/n until achievement of the desired sample size. In consecutive sampling, the first patient was randomly selected, then patients consecutively registered were selected until the achievement of the required sample size.

### Statistical analyses

We first compared estimates of the retention on ART at 12 months obtained using Kaplan-Meier methods (taking into account the exact duration of follow-up of each patient before treatment discontinuation) with the proportion of patients retained on ART at 12 months (as recommended by UNGASS [5]). The outcomes were either death, stopping ART or LFU within the first 12 months, while for patients alive and on ART follow-up was censored at 12 months.

Thereafter all estimates were generated as proportions at 12 months as it is the usual method in routine programme monitoring. We calculated the two indicators using first the overall dataset and compared the proportions with those resulting from computation in sub-samples obtained with the three sampling methods. Indicators obtained using the full dataset are referred to as “unsampled” values. For each site and for each sampling method, 500 samples were simulated. We used the mean of the 500 results and the 2.5^th^ and 97.5^th^ values to determine the 95% sampling distribution (SD) and compared it to the unsampled site value. Indicators obtained using the full dataset were also compared to those obtained with the sampling methods according to cohort size taking the median value (870 patients) as a threshold, type of setting (rural/urban) and the proportion of patients LFU in the cohort (<10% versus ≥10%).

Combined indicators for all sites were then generated by aggregating site specific sampling results; the estimate of the mean was a weighted average of the proportion at each site weighted by the sample proportion (i.e., the number of patients selected divided by the total number of eligible patients at each site). Statistical analyses were performed using Statistical Analysis System software (SAS, version 9.1).

## Results

Twenty-seven ART sites, 22 located in Africa and five in Asia, were included with a total of 27,201 patients treated. The number of patients per site ranged from 378 to 4111. After 12 months on ART 2,036 (7.5%) patients had died (range 1.9% to 16.7% across sites), 688 (2.5%) had stopped ART (range: 0% to 8.5%) and 13.5% were LFU (range 0.8% to 31.9%) (Table 1). Estimates of retention on ART at 12 months calculated with the proportion and Kaplan-Meier methods were similar, at 75.9% (range 58.7% to 88.6%) and 76.5% (58.9% to 88.6%) respectively. All following results were generated as proportions as it is the usual method in routine programme monitoring.

**Table 1:** Number of patients analysed and treatment outcomes at 12 months of ART by cohort on the full dataset.

ART site	Country	Number of adults starting ART in 2005	Number of adults sampled	Number of treatment outcomes at 12 months and proportion among all adults starting ART in 2005							
				Deaths		ART discontinuations		Lost to follow-up ≥90 days from last visit		Retention on ART at 12 months	
		n	n	n	%	n	%	n	%	n	%
Kampong Cham	Cambodia	606	196	58	9.6	29	4.8	27	4.4	492	81.1
Phnom Penh	Cambodia	610	196	20	3.3	52	8.5	5	0.8	533	87.4
Siem Reap	Cambodia	424	172	29	6.8	17	4.0	15	3.5	363	85.6
Takeo	Cambodia	491	182	45	9.2	6	1.2	16	3.2	424	86.3
Pissy	Burkina-Faso	899	219	54	6.0	52	5.8	143	15.9	650	72.3
Kinshasa	DRC	1065	227	178	16.7	14	1.3	86	8.1	787	73.9
Busia	Kenya	860	217	55	6.4	26	3.0	73	8.5	706	82.1
Homabay	Kenya	954	222	101	10.6	15	1.6	104	10.9	734	76.9
Kibera	Kenya	435	174	23	5.3	25	5.7	49	11.3	338	77.7
Mathare	Kenya	549	190	23	4.2	32	5.8	74	13.5	420	76.5
Chiradzulu	Malawi	1599	245	150	9.4	22	1.4	186	11.6	1241	77.6
Thyolo	Malawi	1359	238	172	12.7	33	2.4	121	8.9	1033	76.0
AltoMae	Mozambique	1208	233	23	1.9	22	1.8	245	20.3	918	76.0
Mavalan	Mozambique	1294	236	65	5.0	49	3.8	132	10.2	1048	81.0
Moatize	Mozambique	278	142	15	5.4	14	5.0	12	4.3	237	85.2
Lagos	Nigeria	713	206	41	5.7	12	1.7	69	9.7	591	82.9
Arua	Uganda	1137	231	44	3.9	33	2.9	153	13.4	907	79.8
Kapiri Kawama	Zambia	559	191	67	12.0	5	0.9	19	3.4	468	83.7
Bulawayo	Zimbabwe	953	222	110	11.5	5	0.5	129	13.5	709	74.4
Murambinda	Zimbabwe	428	173	68	15.9	2	0.5	14	3.3	344	80.4
**Total MSF**		**16421**	**4112**	**1341**	**8.2**	**465**	**2.8**	**1672**	**10.2**	**12943**	**78.8**
YRG care	India	767	210	28	3.6	46	6.0	245	31.9	452	58.9
CEPREF	Côte d'Ivoire	1127	230	89	7.9	0	0.0	123	10.9	915	81.2
AMPATH	Kenya	4111	270	277	6.7	157	3.8	618	15.0	3067	74.6
Lighthouse	Malawi	2177	255	179	8.2	0	0.0	643	29.5	1355	62.2
Gugulethu	South Africa	870	217	71	8.2	13	1.5	70	8.0	721	82.9
ISS	Uganda	1350	238	27	2.0	1	0.1	299	22.1	1023	75.8
Connaught	Zimbabwe	378	164	24	6.3	6	1.6	15	4.0	335	88.6
**Total IeDEA**		**10780**	**1584**	**695**	**6.4**	**223**	**2.1**	**2013**	**18.7**	**7868**	**73.0**
**Total**		**27201**	**5696**	**2036**	**7.5**	**688**	**2.5**	**3685**	**13.5**	**20811**	**76.5**

**Figure 1:**
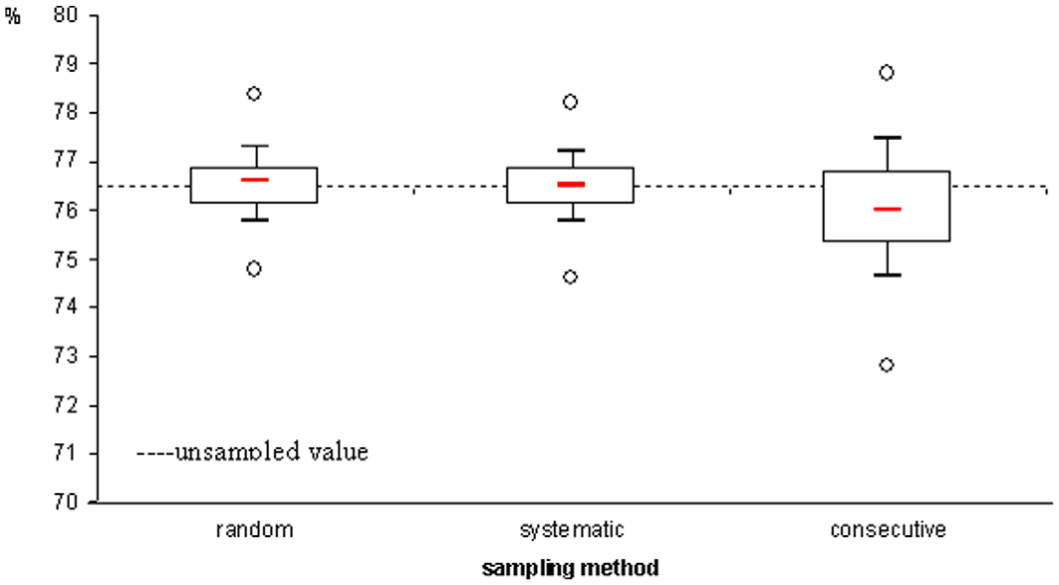
Proportion of patients on ART at 12 months: comparison of estimates according to the sampling technique (median, inter-quartile range, 10–90% deciles and minimum maximum) with the unsampled value (dotted line).

A total of 5,696 patients (20.9%; range 6.6% to 51.1% across cohorts) were sampled. Estimates for 12-month retention on ART, from random, systematic and consecutive sampling were 76.5% (95% SD 75.4–77.7), 76.5% (95% SD 75.3–77.5) and 76.0% (95% SD 74.1–78.2), respectively, compared to 76.5% for the unsampled value (Figure 1). The sample distribution for the 500 sample iterations varied across sites. Overall, sample distribution was wider when using consecutive sampling; the 2.5^th^ value was within minus 5% of the unsampled value for 14/27 sites (51.8%) and the 97.5^th^ value was within plus 5% for 21/27 sites (78%) and ranged from −10.0% to +10.6%. Variability in sample distribution was independent of cohort size (*P* = 0.98), urban/rural location (*P* = 0.99) or the proportion of patients LFU in the cohort (*P* = 0.81).

**Figure 2:**
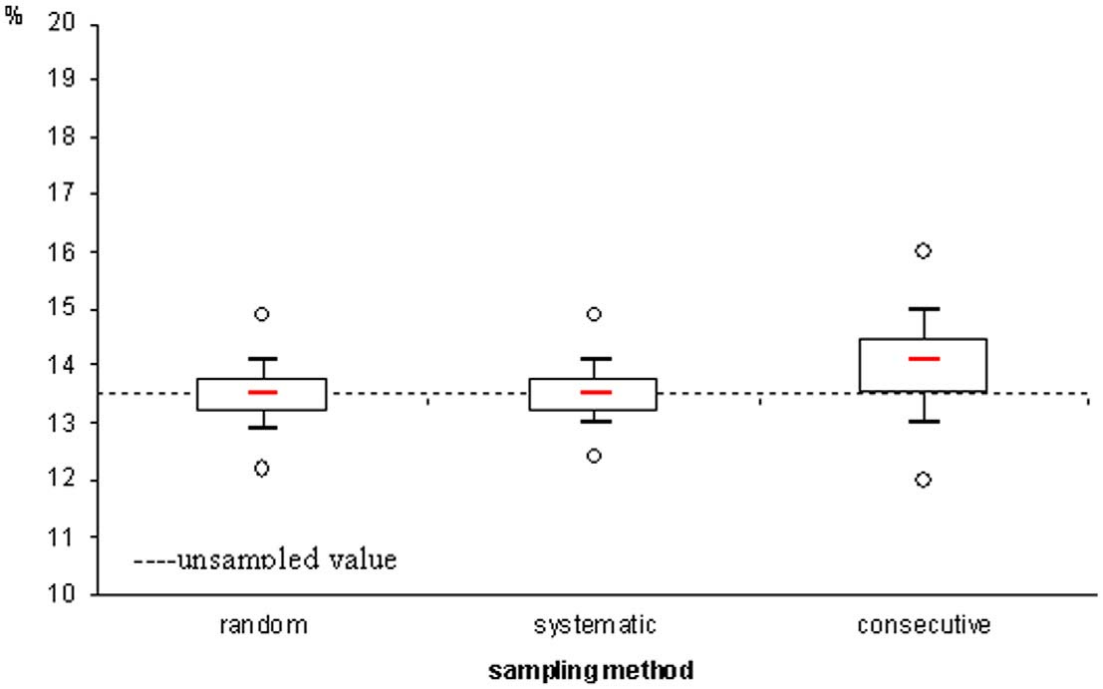
Proportion of patients lost to follow-up at 12 months: comparison of estimates according to the sampling technique (median, inter-quartile range, 10–90% deciles and minimum maximum) with the unsampled value (dotted line).

Similar results were observed for the proportion of patients LFU indicator (Figure 2). The consecutive sampling method slightly overestimated the estimates (14.0%; 95% SD 12.5–15.5) compared to 13.5% using the full dataset, and the sample distribution was wider than intervals obtained with random (13.5%; 95% SD 12.6–14.5) and systematic sampling (13.5%; 95% SD 12.6–14.3).

## Discussion

When paper records are used at ART sites, programme monitoring often relies on transferring key information from patients' medical records into paper registers or electronic databases and aggregating the information at regular periods for reporting and interpretation of findings at all levels of the health system. Maintaining accurate medical records for all patients at every contact is essential to ensure quality of care and patient management, but the subsequent transfer of information for the purpose of programme monitoring could be limited to a sample of patients rather than the full cohort to reduce workload. Sampling has already been used for HIV/AIDS care program monitoring [11] but precision of the results obtained had not been assessed. Sampling has also been used to determine outcomes among patients LFU by tracing in the community [12], [13].

We compared two monitoring indicators, proportions of patients retained on ART and of patients LFU at 12 months. These statistics were obtained first with the overall dataset without sampling, then after applying three sampling strategies. Many international indicators, including the UNGASS indicators, are indeed calculated only at a national level and do not require exhaustive site-specific data. Our sampling strategies performed well on the dataset combining patient data collected in 27 sites. Overall estimates were similar independently of the sampling method used. Sampling performed particularly well when indicators were calculated in the full dataset while differences in estimates and sample distribution were more variable when analysed at site level. For 12-month retention on ART, the sample distribution obtained by consecutive sampling was wider compared to random and systematic sampling methods with a maximum distribution of ±10% of the unsampled value. Whereas not directly comparable, the cluster sampling method used to monitor immunization coverage in children was developed three decades ago for sampling results to range within ±10% from the population value and has always been recommended since then; although 17% of sampling results fell outside these limits, the method was considered to perform well enough for programmatic purposes [14].

Patient life-long retention on ART is of growing concern in the rapid scale-up of large treatment programmes[4], [15]–[17]. An analysis in South Africa showed a deterioration in retention on ART over time with an increasing proportion of patients LFU among those enrolled during the most recent years. It was in part related to the large increase in patients but the authors also discussed the burden on health informatics systems and administrative errors leading to misclassification in LFU [15]. Improving and maintaining simple and standardised monitoring systems capturing true treatment outcomes is part of the strategies recommended to better document and therefore improve patient retention on ART [17].

The workload required to ensure good quality of ART cohort monitoring is substantial for both paper-based and electronic systems. It also increases with the size and follow-up of the cohort. EMR systems allow the systematic and sometimes automated production of statistical indicators using information from all patients, but they are resource-intensive, as they require data clerks, training, and system maintenance[18]. A review of EMR systems used in 21 ART sites in low- and middle-income countries reported a median proportion of missing data for key information of 10.9% [19]. Missing data declined with training on data-management and with the number of hours spent by data-clerks in the maintenance of the databases. The number of hours necessary to reduce the proportion of missing information below 10% was estimated at 10 hours per week per 100 patients on ART. Time is also required to set the system up and expand it throughout a country. Some countries are moving towards a national EMR system; SmartCare is an EMR system currently deployed in Zambia, Ethiopia and South Africa [20]. However, in the current situation where international donors are not providing additional financial support, countries where an insufficient proportion of persons in need of ART are receiving treatment may prioritize employing and training additional clinical and laboratory staff to provide patient care over investing resources to develop and maintain an EMR system.

In the absence of EMR that would support the analyses of full cohort data, sampling approaches could thus potentially limit the workload in longitudinal monitoring and reinforce the long-term sustainability of monitoring systems at local and national levels. Moreover, retention on ART is to be analysed not only at 12 months but also for subsequent years of follow-up to document trends in retention on ART over time. The number of yearly end-points to analyse will therefore increase with the maturity of the programs. Sampling might be particularly useful in ART sites with a large number of patients initiating ART annually. Based on our analysis, the minimum sample size for a cohort of 500 patients would be 184, 224 for a cohort of 1000 and 252 for a cohort of 2000 patients. Consecutive sampling would probably be the most practical approach for the selection of patients. A number of countries are currently collecting retrospective patient data to generate longitudinal indicators once or twice a year; abstraction of information and calculation of indicators could potentially be performed using a sample of patients starting on ART in each calendar year. Sampling could be also piloted as an interim strategy while implementing EMR and/or could be used for purpose of quality control.

The present evaluation was based on existing databases in well resourced sites that receive support to maintain complete, quality-assured medical records. Thus the performance observed for sampling is not directly generalisable to a national programme. Performance of sampling strategies will depend on the accuracy or completeness of medical records and/or registers and on the level of organisation of the filing system. This evaluation did not address the issue of additional support that may be required in many ART sites where data are missing or inaccurately recorded. Sampling may also be difficult to implement in routine monitoring and needs a standardised and sustainable method for the long-term comparability of results. Personnel at site level may not be confident with results produced by sampling. Pilot projects to produce and validate monitoring indicators from sampling and to quantify related workload are therefore needed to complement this work.

In conclusion, generation of the two longitudinal indicators we studied in a sample of patients appeared to be a potentially useful method for programme monitoring at national level, based on available data from the ART-LINC of IeDEA and MSF cohort collaborations. However, the feasibility of this approach needs to be evaluated at country level and the accuracy of estimates based on sampling should be evaluated at site level in countries interested in this approach.
